# Supplementary material for: Chromothripsis during telomere crisis is independent of NHEJ, and consistent with a replicative origin
Source: Genome Res. 2019 May;29(5):737–49. doi: 10.1101/gr.240705.118 (PMC6499312; doi:10.1101/gr.240705.118)
Supplement: Supplemental Material [file supp_gr.240705.118_Supplemental_file_1.zip › contigs/annotated_contigs/DB91/contig.2.DB91_length_323_mean_cov_2.16408668731.docx]

**DB91_length_323_mean_cov_2.16408668731**

```
TTGAGCATGCAGATGCCAAAGATCTAGCCACTGTACATATT|CCTGGAAGACAGAGCAATAAGCTGTCTCATAAGAGAAAAAAAAAACC
                                          >chrY:13295270-13295450 + E=4e-62 p=2e-02
ATAATGCCACCTAATATCCAGTTTATACTTAAATTTCCCTAAGTGTCTGAATAATTTTTTTTTTTGAAACATGGTCTCAACTTTGTCAC

CCAGGATGGAGTGCAGTGTTGCGATCTCAGCTCACTGCAAC|AGCTGAAGCCGGCATACAAGAGATTGACCGGCGACTGCAGTCTGGGC

GGGTGCGCTTCCAGGCCGAGGTCGA|GCCATGGCTGGCTAATTTTTTGTATTTTAGTAA                          >chrUn_gl000211:87351-87384 + E=
```
